# Integrin beta 4 (ITGB4) and its tyrosine-1510 phosphorylation promote pancreatic tumorigenesis and regulate the MEK1-ERK1/2 signaling pathway

**DOI:** 10.17305/bjbms.2019.4255

**Published:** 2020-02

**Authors:** Xiangli Meng, Peng Liu, Yunhao Wu, Xinlu Liu, Yinpeng Huang, Boqiang Yu, Jiahong Han, Haoyi Jin, Xiaodong Tan

**Affiliations:** 1 Department of the First General Surgery, Shengjing Hospital affiliated to China Medical University, Shenyang, China; 2 Department of Anus and Intestine Surgery, The First Affiliated Hospital of Dalian Medical University, Dalian, China; 3 Minimally Invasive Area of General Surgery, The First Affiliated Hospital of Jinzhou Medical University, Jinzhou, China; 4 Department of General Surgery, Fushun Central Hospital, Fushun, China; 5 Department of Surgery, Liaoning Electric Power Center Hospital, Shenyang, China

**Keywords:** ITGB4, integrin, MEK1 (T292), invasion, migration, pancreatic cancer

## Abstract

Pancreatic cancer is the fourth leading cause of cancer death, with a 5-year survival rate of only 1–4%. Integrin-mediated cell adhesion is critical for the initiation, progression, and metastasis of cancer. In this study we investigated the role of integrin β4 (ITGB4) and its phosphorylation at tyrosine Y1510 (p-ITGB4-Y1510) in the tumorigenesis of pancreatic cancer. We analyzed the expression of ITGB4 and p-ITGB4-Y1510 in pancreatic cancer tissue and cell lines using immunohistochemistry, Western blot, or semi-quantitative reverse transcription PCR. ITGB4 and p-ITGB4-Y1510 were highly expressed in pancreatic cancer (n = 176) compared with normal pancreatic tissue (n = 171). High p-ITGB4-Y1510 expression correlated with local invasion and distant metastasis of pancreatic cancer, and high ITGB4 was significantly associated with poor survival of patients. Inhibition of ITGB4 by siRNA significantly reduced migration and invasion of PC-1.0 and AsPC-1 cells. Overexpression of the mutant ITGB4-Y1510A (a mutation of tyrosine to alanine at 1510 position) in PC-1.0 and AsPC-1 cells not only blocked the ITGB4 phosphorylation at Y1510 but also suppressed the expression of ITGB4 (*p* < 0.05 vs. wild-type ITGB4). The transfection of PC-1.0 and AsPC-1 cells with ITGB4-Y1510A significantly decreased the level of p-mitogen-activated protein kinase kinase (MEK)1 (T292) and p-extracellular signal-regulated kinase (ERK)1/2 but did not affect the level of p-MEK1 (T386) and p-MEK2 (T394). Overall, our study showed that ITGB4 and its phosphorylated form promote cell migration and invasion in pancreatic cancer and that p-ITGB4-Y1510 regulates the downstream MEK1-ERK1/2 signaling cascades. Targeting ITGB4 or its phosphorylation at Y1510 may be a novel therapeutic option for pancreatic cancer.

## INTRODUCTION

Although pancreatic cancer accounts for only 1–3% of newly diagnosed cancer patients every year, with a 5-year survival rate of 1–4% it is the fourth leading cause of cancer death [1,2]. Surgery is a relatively effective treatment for pancreatic cancer, especially considering that most patients are in the advanced stage and with different degrees of distant metastasis at the time of treatment. In 70% of the patients who undergo surgical resection, the cancer will relapse and metastasize within one year [3].

In recent years, we have gained a better understanding of the occurrence, development, invasion, and metastasis of pancreatic cancer [4-6]. However, specific molecular targets in pancreatic cancer have not been fully characterized. The identification of the potential targets and a better understanding of the molecular mechanism driving pancreatic tumorigenesis are crucial to develop new strategies for the early diagnosis and treatment of pancreatic cancer [7-9].

Integrin-mediated cell-matrix adhesion between pancreatic cancer cells and the extracellular matrix (ECM) was recently suggested to play an important role in the development of pancreatic cancer [10,11]. Among integrins, integrin β4 (ITGB4) forms heterodimers with integrin α6 to achieve its biological functions. Integrins also interact with receptor tyrosine kinases (RTKs) to regulate downstream signaling pathways. Recent studies showed that ITGB4 has an important role in promoting carcinogenesis in prostate cancer [12], breast cancer [13], gastric cancer [14], and lung squamous-cell carcinoma [15], as well as that ITGB4 is involved in the invasion and metastasis of breast and prostate cancer. In prostate cancer, ITGB4 appears to promote tumorigenesis by enhancing the signaling of the RTKs ErbB2 and c-Met in tumor progenitor cells [12]. In xenografted pancreatic ductal adenocarcinoma (PDAC) cells netrin-1 inhibited the tumorigenicity of cells, presumably by reducing ITGB4 expression via the inhibition of MEK/ERK/c-Jun signaling pathway [12,16]. However, the exact role of ITGB4 in pancreatic tumorigenesis and pancreatic cancer invasion and metastasis remains unclear.

The MAPK/MEK/ERK signaling pathway regulates gene expression, cell proliferation, differentiation, and apoptosis [17,18]. In pancreatic cancer patients, RAS mutations occur in 70–90% patients [19], which results in an abnormal activation of mitogen-activated protein kinase kinase (MEK)1 and MEK2, and subsequent hyperproliferation of pancreatic cancer cells. Activation of MEK1 and MEK2 can rapidly trigger the downstream extracellular signal-regulated kinase (ERK)1/2 and lead to the initiation of hundreds of target genes, including transcription factors, kinases and cytoskeletal proteins – all of which can affect tumor angiogenesis, invasion, and metastasis [20-23]. Furthermore, targeting MEKs can effectively inhibit pancreatic cancer cell growth, block cell cycle changes, and reduce DNA methyltransferase activity [24].

We previously conducted a high-throughput phosphorylation array analysis to identify candidate proteins involved in pancreatic tumorigenesis. ITGB4 phosphorylated at tyrosine site 1510 (p-ITGB4-Y1510) was upregulated more than 2-fold in high-invasive pancreatic cancer cells, suggesting that phosphorylated ITGB4 may be involved in the migration and invasion of pancreatic cancer cells. In the current study, we further investigated the role of ITGB4 and its phosphorylation at Y1510 in the tumorigenesis of pancreatic cancer. We demonstrated that ITGB4 and its phosphorylated form are not only responsible for the regulation of pancreatic cancer cell migration and invasion, but are also associated with poor survival of pancreatic cancer patients. The inhibition of phosphorylation of ITGB4 at Y1510 in pancreatic cancer PC-1.0 and AsPC-1 cells significantly decreased the level of p-MEK1 (T292) and p-ERK1/2 but did not affect the level of p-MEK1 (T386) and p-MEK2 (T394). Our results indicate that ITGB4 and its phosphorylation at Y1510 have a pivotal role in the development of pancreatic cancer, and that p-ITGB4-Y1510 regulates the downstream MEK1-ERK1/2 signaling cascades.

## MATERIALS AND METHODS

### Tissue specimens

A total of 176 specimens of pancreatic cancer tissues and 171 specimens of normal pancreatic tissues were collected at the Department of the First General Surgery of the Shengjing Hospital affiliated to China Medical University from January 2011 to January 2018. The pancreatic tumors were diagnosed postoperatively by pathology. All specimens were collected according to the human specimen collection procedure and approved by the Chinese Ethics Review Committee. Written informed consent was obtained from all patients. The study was conducted in accordance with the guidelines of the Declaration of Helsinki.

For survival analysis, the pancreatic cancer tissues were classified into groups with low (n = 37) and high ITGB4 (n = 139) expression. Moreover, we analyzed p-ITGB4-Y1510 expression in relation to local invasion and distant metastasis of pancreatic cancer.

### Cell culture and transfection

The pancreatic cancer cell lines PC-1.0 and AsPC-1 were established from a N-nitrosobis(2-oxopropyl)amine (BOP)-induced Syrian hamster pancreatic cancer model [25,26]. The cells were seeded in polylysine-coated flasks with a density of 5×10^5^/mL in RPMI-1640 medium (Gibco, Grand Island Biological Company, NY, USA) supplemented with 10% fetal bovine serum (FBS), at 37°C and in an atmosphere of 95% air and 5% CO_2_. For overexpression experiments, cells grown to 70% confluency were transfected with a wild-type (WT)-ITGB4 or ITGB4-Y1510A (a mutation of tyrosine to alanine at 1510 position) plasmid using Lipofectamine 2000 (Invitrogen, Carlsbad, CA, USA). After 6 h of transfection, the culture medium was replaced with fresh medium and the cells were cultured for additional 48 h. For gene silencing by small interfering RNA (siRNA), the cells were transfected with scrambled control siRNA (si-NC) or ITGB4 siRNA [si-ITGB4] (Santa Cruz Biotechnology, Dallas, TX, USA) using an RNAiMAX reagent (Invitrogen), according to the manufacturer’s instructions. In brief, the siRNA oligonucleotides were mixed with RNAiMAX reagent in an Opti-MEM medium (Invitrogen). After 20 min of incubation at room temperature, the cells were incubated with a well-optimized transfection mixture for 24 h at 37°C.

### Cell migration assay

Cell migration ability was analyzed by scratch assay. PC-1.0 and AsPC-1 cells were seeded in 6-well plates at a density of 5×10^5^ per well and allowed to grow overnight, to reach a confluency of approximately 85%. A 200 µL sterile pipette tip was used to draw several straight lines across the cell layer. The cells were washed twice with phosphate-buffered saline (PBS) and cultured in complete medium containing 10% FBS. After 6 and 12 h of incubation, the cells were imaged under a microscope.

### Cell invasion assay

PC-1.0 and AsPC-1 cells were suspended in FBS-free medium with a cell density of 1×10^5^/mL. For the invasion assay, 200 µL of cell suspension was added to the upper chamber of the transwell, and 600 µL of complete medium containing 10% FBS was placed in the lower chamber. After 24 h of incubation, the medium in the lower chamber was aspirated and washed 3 times with PBS. Invasive cells at the surface of lower chamber were fixed with 4% paraformaldehyde for 30 min and stained with 0.1% crystal violet for 30 min. After the upper-chamber cells were wiped off, the cells were imaged under a microscope. A total of six fields were randomly selected and total invasive cells were counted.

### Semi-quantitative reverse transcription polymerase chain reaction (RT-PCR)

The total RNA was extracted from pancreatic normal and cancer tissues by TRIzol reagent (Gibco Invitrogen). For 100 mg sample 1 mL of TRIzol was used to extract the total RNA. The quality and integrity of the extracted RNAs were examined by agarose gel electrophoresis by visualizing the 18S and 28S ribosomal RNA bands. Complementary DNA (cDNA) was synthesized from 1 mg of total RNA using M-MLV reverse transcriptase (Ambion) as previously described [27]. After incubation at 42°C for 2 h, the reaction mixtures were heat inactivated at 70°C for 10 min and then applied as templates to amplify cDNAs specific for ITGB4 (amplified with the forward primer 5’- CTGGAGGTGTTTGAGCCACT-3’ and the reverse primer 5’-TTACAACAGCATTGGTACTTGGAT-3’). Glyceraldehyde 3-phosphate dehydrogenase (GADPH) internal control was amplified using the primer set 5’-TGACTTCAACAGCGACACCCA-3’ and 5’- CACC CTGTTGCTGTAGCCAAA-3’. The relative expression changes of genes were calculated by the 2^−△△CT^ method. The reaction conditions were as follows: pre-denaturation at 95°C for 5 min, then 94°C for 30 s, 55°C for 30 s, and 72°C for 60 s for 30 cycles, and 72°C for 1 min.

### Sodium dodecyl sulfate–polyacrylamide gel electrophoresis (SDS-PAGE) and Western blotting analysis

Western blotting was performed as previously described [28]. Samples from PC-1.0 and AsPC-1 cells were lysed in a lysis buffer containing 0.5 mM phenylmethylsulfonylfluorid (PMSF), phosphatase inhibitors, and protease inhibitor cocktail. After removing the cell debris, protein concentration was analyzed using a bicinchoninic acid (BCA) protein assay and subsequently subjected to electrophoresis in polyacrylamide gels. After transferring the protein to polyvinylidene difluoride (PVDF) membranes, the membranes were blocked with TBS containing 5% w/v nonfat milk and probed with specific primary antibodies. The primary antibodies against ITGB4, p-ITGB4 (Y1510), MEK-1, p-MEK-1 (T292), p-MEK-1 (T386), MEK-2, p-MEK-2 (T394), ERK1/2, and p-ERK1/2 (T202/T204) were purchased from ImmunoWay (1:1000 dilution, Suzhou, China). A control antibody against GAPDH was obtained from Santa Cruz Biotechnology (1:1000 dilution). After incubation for 2 h at room temperature, the membranes were washed with TBS Tween 20 (TBST) for 3 times and probed with secondary antibodies conjugated with horseradish peroxidase [HRP] (Santa Cruz Biotechnology, Dallas, TX, USA, 1:1000) for 1 h at room temperature. The chemiluminescent signal was visualized using a Western Lightning Plus-ECL reagent (PerkinElmer) and the signals were imaged by gel imaging system.

### Immunohistochemistry (IHC)

After routine dewaxing of the tissue sections, the sections were placed in a citrate buffer solution and the antigen was repaired by microwave method, heated in a microwave oven, and cooled down to room temperature. Next, 3% H_2_O_2_ was added dropwise to the tissue samples, incubated at room temperature for 10 min to block endogenous oxidases, and washed in PBS 3 times for 5 min. ITGB4 or p-ITGB4 (Y1510) primary antibodies (1:300) were added, washed in PBS 3 times for 5 min, and then HRP-labeled secondary antibody (1:500, Santa Cruz, USA) was added. The tissue samples were incubated with antibodies for 1 h at room temperature and washed 3 times for 5 min with PBS. DAB (3,3’-diaminobenzidine) was exposed to light for 5 min at room temperature, counterstained for 3 min with hematoxylin, and washed 3 times for 5 min with PBS. Dehydrated, xylene transparent, and neutral gum seals were prepared. Six sections of specimens from normal and tumor groups were selected, and the protein expression in each group was observed under a light microscope. The optical density values were analyzed and measured using Image-Pro Plus 6.0 software.

### Statistical analysis

All experiments were performed at least in three independent replicates. The results are presented as mean ± standard deviation (mean ± SD). Statistical analysis was performed using SPSS Statistics for Windows, Version 17.0. (SPSS Inc., Chicago, USA). The significance of the differences between the experimental groups was analyzed by one-way ANOVA. The cumulative survival curve was estimated by the Kaplan–Meier method. The *p* values are denoted with asterisks: *p < 0.05, **p < 0.01, and ***p < 0.001. In this study *p* < 0.05 was considered statistically significant.

## RESULTS

### ITGB4 is highly expressed in pancreatic cancer tissues and is associated with poor survival of patients

**FIGURE 1:**
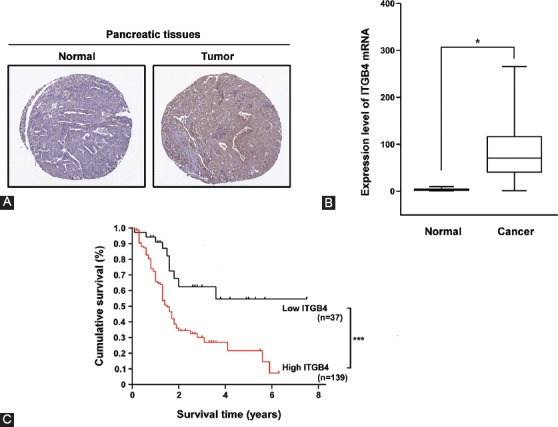
ITGB4 was highly expressed in pancreatic cancer tissues and associated with poor survival of patients. (A) Immunohistochemical analysis of ITGB4 expression in normal pancreatic and pancreatic cancer tissues. (B) Quantitative analysis of ITGB4 expression in normal vs. pancreatic cancer tissues by semi-quantitative RT-PCR. The results are expressed as mean ± SD, and differences were considered statistically significant when *p < 0.05. (C) Kaplan–Meier analysis of overall survival rates among 176 patients with pancreatic cancer that were classified in low ITGB4 and high ITGB4 expression group. High expression of ITGB4 was significantly correlated with poorer overall survival of patients (***p < 0.05). ITGB4: Integrin β4; RT-PCR: Reverse-transcription polymerase chain reaction.

In our previous study, we found that ITGB4 was highly expressed in high-invasive metastatic pancreatic cancer cell line PC-1.0 compared to low-invasive cell line PC-1 [29], implying that ITGB4 may be functionally involved in the tumorigenicity of pancreatic cancer. Thus, in this study, we first examined whether ITGB4 was highly expressed in 176 specimens of pancreatic cancer tissues compared with 171 specimens of normal pancreatic tissues. The IHC analysis showed that the expression of ITGB4 was highly increased in pancreatic cancer tissues (Figure 1A). The significantly higher expression of ITGB4 in pancreatic cancer vs. normal tissues was further confirmed by semi-quantitative RT-PCR (Figure 1B, *p* < 0.05). We then estimated the prognostic value of ITGB4 in patients with pancreatic cancer. The estimated 5-year overall survival rates among 176 patients were 55% and 22% for the low ITGB4 and high ITGB4 expression group, respectively. The expression of ITGB4 was significantly correlated with poorer overall survival of patients (Figure 1C, *p* < 0.001).

### The role of ITGB4 in migration and invasion of pancreatic cancer cells

**FIGURE 2:**
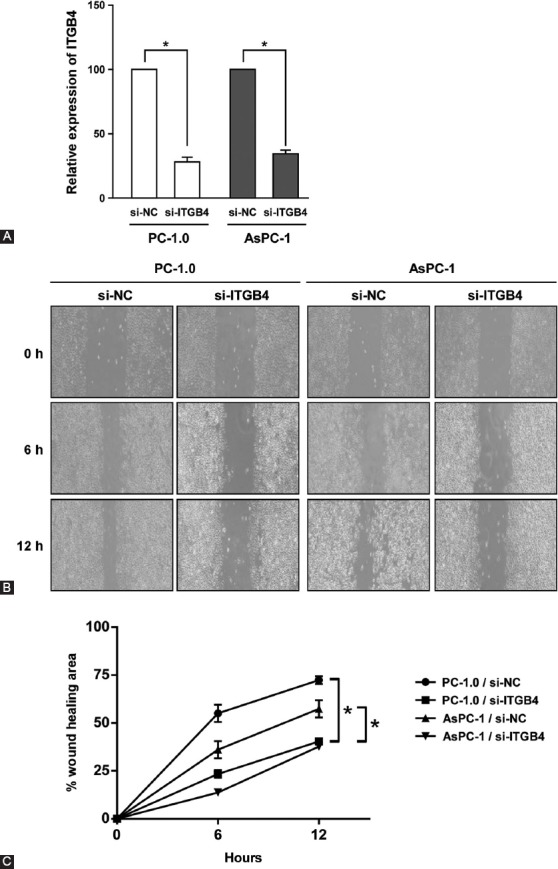
ITGB4 promotes migration of pancreatic cancer cells. (A) After siRNA knockdown of ITGB4 in PC-1.0 and AsPC-1 cells, the relative expression of ITGB4 was determined by Western blotting. (B) Scratch assay of PC-1.0 and AsPC-1 cells upon the inhibition of ITGB4 expression. (C) Quantification of the migration ratio of PC-1.0 and AsPC-1 cells treated with si-NC or si-ITGB4. The error bars represent the standard deviation of three independent experiments. p < 0.05 was considered statistically significant. ITGB4: Integrin β4; siRNA: Small interfering RNA; si-NC: Scrambled control siRNA; si-ITGB4: ITGB4 siRNA.

To further explore the biological function of ITGB4 in pancreatic cancer, we analyzed cell migration by scratch assay in pancreatic cell lines PC-1.0 and AsPC-1. The cells were first transfected with si-NC or si-ITGB4 for 24 h, and the knockdown effect was examined by Western blotting. Compared with si-NC, si-ITGB4 significantly inhibited ITGB4 expression, to 27% in PC-1.0 and 33% in AsPC-1 cells (Figure 2A, *p* < 0.05). The cell monolayers with si-NC or si-ITGB4 were then scratched with pipette tip. The scratch assay showed a significant reduction in the migration ability of PC-1.0 and AsPC-1 cells transfected with si-ITGB4 compared with si-NC groups (Figure 2B and 2C, *p* < 0.05). While PC-1.0 and AsPC-1 cells transfected with si-NC almost healed the wounds after 12 h, the healed areas of si-ITGB4-transfected cells were less than half of those in the control group.

**FIGURE 3:**
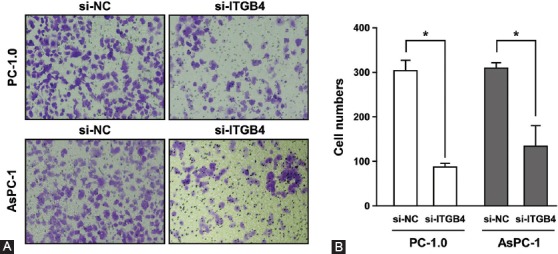
ITGB4 promotes invasion of pancreatic cancer cells. (A) After the treatment of PC-1.0 and AsPC-1 cells with si-NC or si-ITGB4, the invasive ability of cells was determined by transwell assay. Images of invasive cells are shown. (B) Quantitative analysis of the transwell assay results. The cell number passing through the filter in si-ITGB4 group was significantly lower than that in si-NC group. The error bars represent the standard deviation of three independent experiments. p < 0.05 was considered statistically significant. ITGB4: Integrin β4; siRNA: Small interfering RNA; si-NC: Scrambled control siRNA; si-ITGB4: ITGB4 siRNA.

Next, the transwell assay was conducted to analyze the effect of ITGB4 on the invasion ability of pancreatic cancer cells. Consistent with the scratch assay, PC-1.0 and AsPC-1 cells transfected with si-ITGB4 were much less invasive (Figure 3A), with a significant difference between si-NC and si-ITGB4 transfected groups (Figure 3B, *p* < 0.05). In summary, these results indicate that ITGB4 plays an important role in the migration and invasion ability of pancreatic cancer cells.

### Phosphorylation of ITGB4 at Y1510 is associated with the tumorigenicity of pancreatic cancer

**FIGURE 4:**
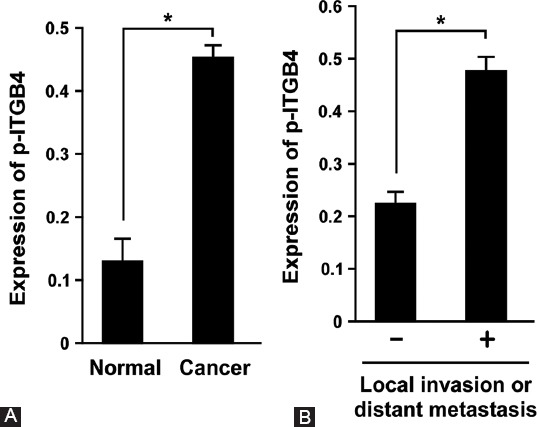
Phosphorylation of ITGB4 at tyrosine 1510 site was associated with the tumorigenicity of pancreatic cancer. (A) The expression of p-ITGB4-Y1510 in normal pancreatic and pancreatic cancer tissues was determined by IHC. The level of p-ITGB4-Y1510 was significantly increased in 176 pancreatic cancer tissues vs. 171 normal pancreatic tissues. (B) The expression level of p-ITGB4-Y1510 was examined in normal tissues vs. local invasion or distant metastasis pancreatic tissues by IHC. The level of p-ITGB4-Y1510 was significantly increased in local invasion/distant metastasis group. *p < 0.05. ITGB4: Integrin β4; p-ITGB4-Y1510: ITGB4 phosphorylated at tyrosine site 1510; IHC: Immunohistochemistry.

Since the phosphorylation of integrin is important for its activity, we analyzed whether the phosphorylation of ITGB4 at Y1510 was increased in pancreatic cancer tissues. The level of p-ITGB4-Y1510 was significantly increased in 176 pancreatic cancer tissues vs. 171 normal pancreatic tissues (Figure 4A, *p* < 0.05). To further investigate whether the phosphorylation of ITGB4 was associated with the tumorigenicity of pancreatic cancer, we analyzed p-ITGB4-Y1510 expression in the tissue specimens from local invasion and distant metastasis and compared with the expression of p-ITGB4-Y1510 in normal tissues. Consistent with the above results, the level of p-ITGB4-Y1510 was significantly increased in local invasion and distant metastasis pancreatic tissues (Figure 4B, *p* < 0.05).

**FIGURE 5:**
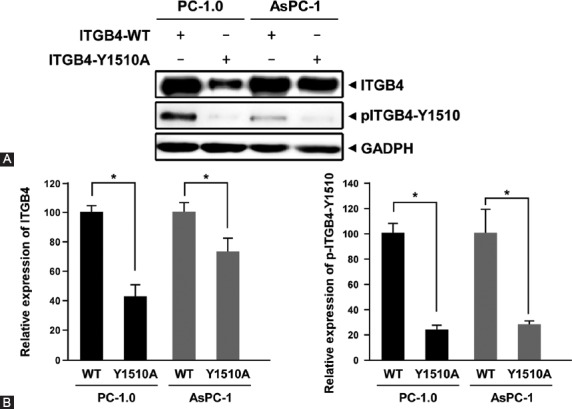
A mutation of tyrosine to alanine (Y1510A) at the position 1510 of ITGB4 reduced ITGB4 expression and eliminated the phosphorylation of ITGB4. (A) PC-1.0 and AsPC-1 cells were transfected with a plasmid containing wild-type (WT)-ITGB4 or ITGB4 with Y1510A mutation and the expression of ITGB4 and p-ITGB4-Y1510 was determined by Western blotting. (B) Quantitative analysis of ITGB4 and p-ITGB4-Y1510 is shown. The expression of p-ITGB4-Y1510 was significantly reduced, to 24% in PC-1.0 and 28% in ASPC-1 cells. The error bars represent the standard deviation of three independent experiments. *p < 0.05 was considered statistically significant. ITGB4: Integrin β4; p-ITGB4-Y1510: ITGB4 phosphorylated at tyrosine site 1510.

To further confirm the role of ITGB4 phosphorylation at Y1510 in ITGB4-mediated cell signaling in pancreatic cancer, we introduced a mutation of tyrosine to alanine at the position 1510 (Y1510A) of ITGB4. This mutation abolishes the tyrosine phosphorylation of ITGB4. PC-1.0 and AsPC-1 pancreatic cells were transfected with WT-ITGB4 or ITGB4-Y1510A plasmid and the expression level of ITGB4 and p-ITGB4-Y1510 was determined by Western blotting. Compared to WT-ITGB4, ITGB4-Y1510A not only reduced the level of p-ITGB4-Y1510 but also inhibited the expression of ITGB4 in both PC-1.0 and AsPC-1 cells (Figure 5A). The level of ITGB4 was normalized to GADPH and the level of p-ITGB4 was normalized to ITGB4. As shown in Figure 5B, the expression level of p-ITGB4-Y1510 was significantly reduced, to 24% in PC-1.0 and 28% in AsPC-1 cells (*p* < 0.05). The reduced expression of ITGB4 in PC-1.0 and AsPC-1 cells transfected with ITGB4-Y1510A suggests that the inhibition of ITGB4 phosphorylation at Y1510 negatively regulates the expression of ITGB4.

### Phosphorylation of ITGB4 at Y1510 regulates the MEK1-ERK1/2 pathway

**FIGURE 6:**
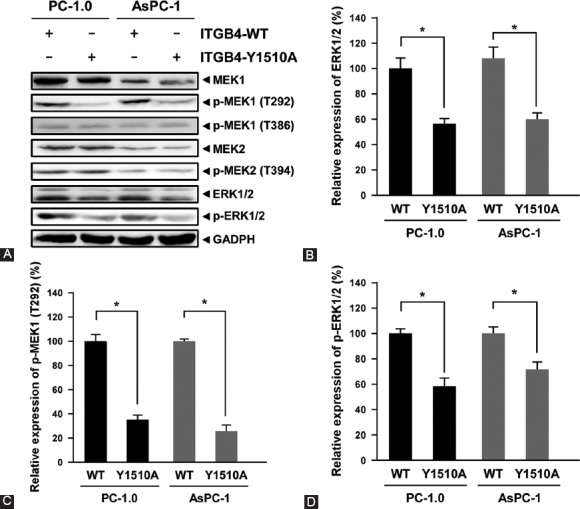
Phosphorylation of ITGB4 at tyrosine 1510 regulated MEK1-ERK1/2 signaling pathway. (A) PC-1.0 and AsPC-1 cells were transfected with a plasmid containing wild-type (WT)-ITGB4 or ITGB4 with Y1510A mutation, and the expression of MEK1, MEK2, ERK1/2, p-MEK1 (T292), p-MEK1 (T386), p-MEK2 (T394), and p-ERK1/2 was determined by Western blotting. (B-D) Quantitative analysis of the relative expression of ERK1/2, p-MEK1 (T292), and p-ERK1/2. The error bars represent the standard deviation of three independent experiments. p < 0.05 was considered statistically significant. ITGB4: Integrin β4; MEK: Mitogen-activated protein kinase kinase; ERK: Extracellular signal–regulated kinase; MAPK: Mitogen-activated protein kinase; p: Phosphorylated.

Since the activation of mitogen-activated protein kinase (MAPK) cascades has shown to play a key role in pancreatic cell proliferation, differentiation, migration, senescence, and apoptosis [30,31] as well as in the tumorigenesis of pancreatic cancer, we next examined whether the phosphorylation of ITGB4 at Y1510 is involved in the regulation of MAPK signaling cascades. PC-1.0 and AsPC-1 cells were transfected with WT-ITGB4 or ITGB4-Y1510A plasmid, and the expression of MEK1, MEK2, ERK1/2, p-MEK1 (T292), p-MEK1 (T386), p-MEK2 (T394), p-ERK1/2 and GADPH were examined by Western blotting (Figure 6A). Compared with WT-ITGB4, ITGB4-Y1510A transfection did not affect the expression of MEK1 and MEK2 but downregulated the expression of ERK1/2 (Figure 6B, *p* < 0.05). Interestingly, the expression level of p-MEK1 (T292) and p-ERK1/2 was significantly decreased in ITGB4-Y1510A-transfected PC-1.0 and AsPC-1 cells (Figure 6C and 6D, *p* < 0.05). However, ITGB4-Y1510A transfection did not affect the level of p-MEK1 (T386) and p-MEK2 (T394). Taken together, these results indicate that ITGB4 not only functionally participates in the migration and invasion of pancreatic cancer cells, but it also regulates the MEK1-ERK1/2 signaling cascade through its phosphorylation at Y1510.

### DISCUSSION

In our previous study, we used the Phospho Explorer antibody array to identify candidate proteins that regulate the invasive ability of pancreatic cancer cells [29]. We found that at least 32 phosphorylated proteins were highly increased in high-invasive metastatic pancreatic cells. In particular p-ITGB4, a member of the integrin family, was increased at least 2-fold in high-invasive vs. weak-invasive pancreatic cancer cells [29]. ITGB4 was reported to be involved in cell death, autophagy, angiogenesis, senescence, and differentiation and to regulate the development of different cancers and diseases of the nervous system [32,33]. However, the role of ITGB4 in pancreatic tumorigenesis remains unclear. Also, the biological role of ITGB4 tyrosine phosphorylation at 1510 site is not clear. In the current study, we aimed to determine whether ITGB4 is involved in pancreatic tumorigenesis and invasion/migration of pancreatic cancer cells as well as to elucidate the molecular mechanism of p-ITGB4-Y1510 in pancreatic cancer. We found that both ITGB4 and p-ITGB4-Y1510 were highly expressed in pancreatic cancer tissues, and the higher ITGB4 expression was significantly associated with poor survival of patients with pancreatic cancer. The inhibition of ITGB4 expression by siRNA indeed suppressed pancreatic cancer cell migration and invasion, indicating that ITGB4 plays a pivotal role in the malignancy of pancreatic cancer.

To achieve its biological functions, ITGB4 associates with integrin α6 and forms α6β4 heterodimers. Integrin α6 has been associated with cancer progression [34-36], including pancreatic cancer [37]. This suggests that ITGB4 may interact with integrin α6 or function independently to trigger downstream signaling cascades involved in pancreatic cancer development, invasion, and metastasis. A recent study demonstrated that annexin A7 (ANXA7) triggers ITGB4 phosphorylation at Y1494 and promotes ITGB4 nuclear translocation to activate the expression of activating transcription factor 3 (ATF3) and a series of downstream genes [38,39]. This indicates that the phosphorylation of ITGB4 plays an important role in its functional independence and effects on downstream signaling.

The phosphorylation of ITGB4 affects its biological function and cellular localization. ITGB4 phosphorylation on the residues S1356 and S1364 plays important roles in the formation and/or stability of hemidesmosomes [40]. T1736 phosphorylation leads to the disruption of the binding site on the plakin domain of plectin [40-42]. Tyrosine phosphorylation of ITGB4 at Y1440, Y526, Y1640, and Y1422 is associated with the expression of inflammatory cytokines [43]. Ionizing radiation (IR) triggers the phosphorylation of ITGB4 at Y1510 and subsequently leads to the activation of the integrin α6β4-Src-AKT signaling pathway [44]. However, it remains unclear whether the phosphorylation of ITGB4 at Y1510 is involved in the tumorigenicity of pancreatic cancer. Here, we revealed that the level of p-ITGB4-Y1510 was significantly increased in pancreatic cancer tissues (Figure 4A) as well as in pancreatic cancer specimens from local or distant metastasis (Figure 4B). Interestingly, the transfection of PC-1.0 and AsPC-1 cells with ITGB4-Y1510A plasmid not only inhibited the level of p-ITGB4-Y1510 but also reduced the expression of ITGB4. This implies that there was a feedback regulatory mechanism at the Y1510 site of ITGB4 or that the phosphorylation of ITGB4 at Y1510 activates ITGB4. Similar results were observed in IR-treated A549 cells [44]. In A549 cells exposed to 6 Gr IR, the expression of ITGB4 was decreased but the level of p-ITGB4-Y1510 was increased. Whether p-ITGB4-Y1510 is involved in a feedback regulation of the integrin expression warrants further investigation.

The integrin α6β4 can function as a laminin receptor [45], an important component of the ECM that affects the adhesion, spread, migration, invasion, proliferation, and apoptosis of cells. In addition, the interaction of ITGB4 with plectin is necessary to establish a connection between the ECM and intermediate filaments. ITGB4 inactivation results in pyloric atresia associated with junctional epidermolysis bullosa [46]. During cellular adhesion, the interaction between ITGB4 and the ECM activates the MAPK signal transduction pathway, playing an important role in the progression of pancreatic cancer. The inhibition of the MAPK signaling pathway suppresses the proliferation and invasion in choriocarcinoma [47], hepatocellular carcinoma [48], breast cancer [49] as well as in pancreatic cancer [50,51]. Remarkably, our study identified that the phosphorylation of ITGB4 at Y1510 is involved in the regulation of the MAPK-MEK1-ERK1/2 signaling pathway (Figure 6). The mutation of ITGB4-Y1510 in PC-1.0 and AsPC-1 cells reduced the level of p-MEK1 (T292) and p-ERK1/2, but the levels of p-MEK1 (T386) and p-MEK2 (394) were not altered. T292 is located in the proline-rich segment of the protein kinase domain of MEK1, and T386 is located in the C-terminal tail of MEK1 [52]. T292 phosphorylation of MEK1 decreases its S298 phosphorylation, which is catalyzed by p21-activated kinase-1 (PAK1). On the other hand, cyclin-dependent kinase (Cdk)5 and ERK1 phosphorylate MEK1 at T386 regulating its kinase activity [52,53]. However, Eblen et al. demonstrated that ERK-mediated feedback phosphorylation of MEK1 at T386 has only a minor effect on the inhibition of MEK1 S298 phosphorylation by ERK and that excess PAKs may overcome the inhibition of S298 phosphorylation caused by ERK-mediated MEK1 T292 phosphorylation [54]. Taken together, these and our results suggest that T292 and T386 phosphorylation in MEK1 play different roles in the downstream signaling cascades. In addition, the phosphorylation of ITGB4 at Y1510 may affect pancreatic tumorigenesis by regulating T292 MEK1 phosphorylation rather than the T386 phosphorylation.

MAPKs are involved in many physiological and pathological processes, and its downstream kinases ERK1/2 can act as a mediator or master regulator of the G1 to S phase transition [55]. Activation/inactivation of ERK1/2 can regulate proliferative genes throughout the G1 phase [56], which influence cell proliferation, differentiation, and survival. In tumor cells, ERK overactivation affects the downstream transcription factors and regulates the expression of certain oncogenes, thus playing a role in tumor invasion and metastasis [24,57]. The results of our study showed that after the inhibition of ITGB4 phosphorylation at Y1510 the level of p-ERK1/2 was significantly decreased (Figure 6D), implying that ERK1/2 promotes the invasion and metastasis of pancreatic cancer cells via the MAPK/MEK/ERK signaling pathway. To validate this hypothesis, we used the MEK inhibitor U0126 to block the MAPK/MEK/ERK signaling pathway in PC-1.0 and AsPC-1 cells. As expected, the relative level of p-MEK1 (T292), p-MEK1 (T386), p-MEK2 (T394), and p-ERK1/2 in the cells was decreased upon the treatment with U1026. Furthermore, the migration and invasion ability of U0126-treated PC-1.0 and AsPC-1 cells was significantly reduced (data not shown).

## CONCLUSION

The present study revealed that ITGB4 plays a crucial role in the tumorigenesis of pancreatic cancer. The levels of ITGB4 and p-ITGB4-Y1510 were highly increased in pancreatic cancer tissues and correlated with local invasion/distant metastasis. Furthermore, the high ITGB4 expression was negatively correlated with the overall survival of the patients. siRNA silencing of ITGB4 expression inhibited the migration and invasion abilities of pancreatic cancer cells. Mutational analysis demonstrated that the phosphorylation of ITGB4 at Y1510 was involved in the MEK1 (T292)-ERK1/2 signaling cascade. Overall, our study not only revealed the pivotal role of ITGB4 and its Y1510 phosphorylation in the tumorigenesis of pancreatic cancer but it also provided a rationale for targeting ITGB4 or its Y1510 phosphorylation, as a novel therapeutic option for pancreatic cancer.
